# The health system impact of false positive newborn screening results for medium-chain acyl-CoA dehydrogenase deficiency: a cohort study

**DOI:** 10.1186/s13023-016-0391-5

**Published:** 2016-02-03

**Authors:** Maria D. Karaceper, Pranesh Chakraborty, Doug Coyle, Kumanan Wilson, Jonathan B. Kronick, Steven Hawken, Christine Davies, Marni Brownell, Linda Dodds, Annette Feigenbaum, Deshayne B. Fell, Scott D. Grosse, Astrid Guttmann, Anne-Marie Laberge, Aizeddin Mhanni, Fiona A. Miller, John J. Mitchell, Meranda Nakhla, Chitra Prasad, Cheryl Rockman-Greenberg, Rebecca Sparkes, Brenda J. Wilson, Beth K. Potter, on behalf of the Canadian Inherited Metabolic Diseases Research Network

**Affiliations:** School of Epidemiology, Public Health and Preventive Medicine, University of Ottawa, 451 Smyth Rd, Ottawa, ON K1H 8 M5 Canada; Newborn Screening Ontario, Children’s Hospital of Eastern Ontario, Ottawa, Ontario Canada; Ottawa Hospital Research Institute, Ottawa, Ontario Canada; Department of Pediatrics, Division of Clinical & Metabolic Genetics, The Hospital for Sick Children, University of Toronto, Toronto, Ontario Canada; Manitoba Centre for Health Policy, Department of Community Health Sciences, College of Medicine, Faculty of Health Sciences, University of Manitoba, Winnipeg, Manitoba Canada; Departments of Obstetrics & Gynecology and Pediatrics, Dalhousie University, Halifax, Nova Scotia Canada; Better Outcomes Registry & Network (BORN) Ontario, Ottawa, Ontario Canada; Centers for Disease Control and Prevention, National Center on Birth Defects and Developmental Disabilities, Atlanta, Georgia USA; Institute for Clinical Evaluative Sciences, Toronto, Ontario Canada; Department of Pediatrics, Division of Paediatric Medicine, The Hospital for Sick Children, University of Toronto, Toronto, Ontario Canada; Medical Genetics, CHU Sainte-Justine and Department of Pediatrics, Université de Montréal, Montréal, Québec Canada; Department of Paediatrics and Child Health, College of Medicine, Faculty of Health Sciences, University of Manitoba, Winnipeg, Manitoba Canada; Institute of Health Policy, Management and Evaluation, University of Toronto, Toronto, Ontario Canada; Montreal Children’s Hospital, McGill University, Montreal, Quebec Canada; Genetics, Metabolism and Pediatrics, London Health Sciences Centre, Western University, London, Ontario Canada; Department of Paediatrics, Section of Clinical Genetics, Alberta Children’s Hospital, Calgary, Alberta Canada; Department of Medicine, University of Ottawa, Ottawa, Ontario Canada

**Keywords:** Neonatal screening, Metabolic diseases, Health services utilization

## Abstract

**Background:**

There is no consensus in the literature regarding the impact of false positive newborn screening results on early health care utilization patterns. We evaluated the impact of false positive newborn screening results for medium-chain acyl-CoA dehydrogenase deficiency (MCADD) in a cohort of Ontario infants.

**Methods:**

The cohort included all children who received newborn screening in Ontario between April 1, 2006 and March 31, 2010. Newborn screening and diagnostic confirmation results were linked to province-wide health care administrative datasets covering physician visits, emergency department visits, and inpatient hospitalizations, to determine health service utilization from April 1, 2006 through March 31, 2012. Incidence rate ratios (IRRs) were used to compare those with false positive results for MCADD to those with negative newborn screening results, stratified by age at service use.

**Results:**

We identified 43 infants with a false positive newborn screening result for MCADD during the study period. These infants experienced significantly higher rates of physician visits (IRR: 1.42) and hospitalizations (IRR: 2.32) in the first year of life relative to a screen negative cohort in adjusted analyses. Differences in health services use were not observed after the first year of life.

**Conclusions:**

The higher use of some health services among false positive infants during the first year of life may be explained by a psychosocial impact of false positive results on parental perceptions of infant health, and/or by differences in underlying health status. Understanding the impact of false positive newborn screening results can help to inform newborn screening programs in designing support and education for families. This is particularly important as additional disorders are added to expanded screening panels, yielding important clinical benefits for affected children but also a higher frequency of false positive findings.

## Background

Population-based newborn screening programs aim to pre-symptomatically identify newborns with treatable rare conditions. Many such programs throughout the world have expanded within the last decade [1], yielding important clinical benefits to affected children [2]. Despite high specificities associated with newborn screening tests, their positive predictive values are often relatively low due to the low birth prevalence of the screened diseases [3]. The expansion of newborn screening panels has consequently resulted in an overall increased number of false positive results.

Published evidence is inconsistent regarding the potential impact of false positive newborn screening results on parental psychosocial experiences [4–9]. Regarding health services use, some authors have observed higher rates of emergency department visits or hospitalizations among infants with false positive screening results, relative to screen-negative controls [7], yet others have found no significant differences [3–5], or differences only among preterm infants [10]. It is challenging to ascribe any increases in health services use to the parental psychosocial impact of receiving a false positive result. Infants born preterm or with low birth weight are more likely to receive positive newborn screening results in the absence of disease, due to their underlying biology and the nature of the biochemical markers used in the screening tests [10–12]. Abnormal values for such markers may also reflect other factors related to infant metabolism. Thus, increased health services use might be attributable to health needs rather than the screening result.

It has been hypothesized that false positive screening results for life-threatening illnesses such as medium-chain acyl-CoA dehydrogenase deficiency (MCADD) might provoke a stronger parental psychosocial response relative to other diseases [10]. MCADD is an inherited metabolic disease included on newborn screening panels in many jurisdictions [13, 14]. It affects the beta-oxidation pathway for medium-chain fatty acids, critical in times of physiological stress such as illness and fasting. Children diagnosed with MCADD have a hindered response to such stress [14, 15] and are susceptible to potentially fatal metabolic crises [16]. However, if early diagnosis is established, prognosis is excellent [14, 15, 17, 18].

Newborn Screening Ontario coordinates newborn bloodspot screening for approximately 140 000 babies born in Ontario, Canada each year. MCADD was added to the panel in April, 2006 [19]. All legal residents of Ontario are eligible for universal health insurance through the Ontario Health Insurance Plan (OHIP), encompassing a range of medically necessary services. At the Institute for Clinical Evaluative Sciences, encoded health care administrative data can be securely linked to newborn screening data with near complete population coverage [20, 21]; these data include information on gestational age and birth weight. This presents a unique opportunity to investigate the impact of a false positive newborn screening result while adjusting for important confounders. The objective of this study was to evaluate whether children who received false positive newborn screening results for MCADD experienced higher rates of health services use in early life relative to a screen negative cohort in Ontario.

## Methods

This study was approved by the Ottawa Health Science Network Research Ethics Board, Protocol # 20120229-01H. Individual participant consent was not sought because the study involved the secondary use of population-wide health care administrative data.

### Study population and data sources

Our source population included all children born in Ontario who received newborn screening between April 1, 2006 and March 31, 2010. The false positive cohort included children who received a positive newborn screening result for MCADD but were ultimately determined to be unaffected. In Ontario, screening for MCADD uses an algorithm involving C8 acylcarnitine (octanoylcarnitine) as a primary analyte, with C6 (hexanoylcarnitine), C10 (decanoylcarnitine), C10:1 (decenoylcarnitine), C8/C10 ratio, and C8/C2 ratio as secondary markers [19]. Infants who screen positive (potentially affected) are referred to one of five regional Newborn Screening Treatment Centres, which are based at pediatric tertiary care centres in the province. The Treatment Centre works with the infant’s primary health care provider to contact the parents and arrange for diagnostic evaluation, and is also responsible for on-going follow-up and management for affected children. The diagnostic evaluation typically includes plasma acylcarnitine profiling, urine organic acid analysis, and testing for mutations in *ACADM* [19]. Neonates are classified by the Treatment Centre and Newborn Screening Ontario medical staff review results. Infants are classified as true positive if they have a disease-associated genotype (e.g., homozygous for the c.985A > G mutation), and/or have persistent abnormal plasma acylcarnitines, and/or hexanoylglycine detected on urine organic acids analysis. Infants with normal metabolic profiles on diagnostic testing are defined as having received false positive screening results.

A primary comparison cohort included all children with negative newborn screening results for all disorders during the same time period. A secondary comparison cohort included 10 controls with negative screening results matched to each child with a false positive result for MCADD, based on sex, calendar year of birth, urban-rural status of the child’s residence, and an area-based indicator of socioeconomic status. The purpose of the matched comparison cohort was to address potential residual confounding by factors that may be associated with access to health services. Individuals were excluded from the study if they were ineligible for health care coverage at the time of birth or deceased within 24 h following birth (a bloodspot sample must be collected at > 24 h of age in Ontario to be considered satisfactory).

Newborn screening diagnostic confirmation data were securely linked to the provincial health care patient registry at the Institute for Clinical Evaluative Sciences, and then to administrative databases encompassing health service visits from April 1, 2006 through March 31, 2012. Physician encounters were identified using the OHIP Claims Database, which captures services provided by Ontario physicians who bill OHIP on a fee-for-service basis; and services provided by most other Ontario physicians who work in capitation payment models [22]. Emergency department (ED) visit data were retrieved from the Canadian Institute for Health Information’s National Ambulatory Care Reporting System, covering nearly all ED visits in Ontario [20]. Inpatient hospitalization data were obtained from the Canadian Institute for Health Information’s Discharge Abstract Database, covering all acute inpatient facilities in the province [21].

### Potential confounding variables

Additional variables were ascertained from the hospitalization database at the time of birth (sex, birth weight, gestational age, season of birth), and Census/geographic data linked by the child’s postal code (socioeconomic status, urban-rural status). We grouped children into low (< 2500 g) and normal/high (≥ 2500 g) birth weight categories. We dichotomized gestational age to preterm (< 37 weeks) and term/post-term (≥ 37 weeks). Season of birth was categorized as January–April, May–August, or September–December.

A proxy measure of socioeconomic status was defined as the neighborhood-level income quintile, based on average household income data from the 2006 Canadian Census, linked to a child’s residential postal code at birth [23]. Neighborhoods were Census “dissemination areas”, with populations of approximately 400–700 persons; income quintiles were assigned across dissemination areas within larger regions [24]. We merged the two lowest and three highest quintiles to define lower and higher socioeconomic status. Urban-rural status of the child’s residence at birth was defined using the Rurality Index for Ontario, based on population size and density and on travel time to higher levels of hospital care [25]. We defined a rural community using a score of ≥ 40, the criterion used for rural physician eligibility in Ontario [26].

### Utilization outcomes

We included each original health service encounter within the study period (physician visits, ED visits, and hospitalizations). If a child had multiple billed procedures within a single physician visit, these were considered as one visit. However, if a child saw multiple physicians on the same day, these were considered separate encounters. Each ED visit was a separate encounter as was each inpatient hospitalization.

### Statistical analysis

Study datasets were linked using unique encoded identifiers and analyzed at the Institute for Clinical Evaluative Sciences; cell sizes < 6 were not reported due to privacy policies. Counts and percentages were calculated and chi-square tests used to examine bivariate associations between sociodemographic characteristics and cohort membership.

The number of physician visits, ED visits, and hospitalizations during the study period were summed for each child. The length of follow-up for each individual was the time elapsed between the date of birth and the earliest of 3 possible end points: the date of OHIP eligibility loss (mainly related to emigration from Ontario), the date of death, or the last date of follow-up for the study. Unadjusted visit rates and incidence rate ratios (IRR) were calculated to compare the false positive and screen negative cohorts.

Using the Vuong test as a criterion [27], we chose negative binomial regression modeling to compute IRRs for health services comparing the false positive with the screen negative cohorts while adjusting for confounders. Influential observations were identified [28, 29] and truncated to the 99^th^ percentile for each service type. Models were stratified by age at the time of visit (< 1 year of age and ≥ 1 year of age). As a sensitivity analysis to address potential residual confounding by premature birth, we re-ran final models restricted to children with term births (≥ 37 weeks’ gestation). Finally, as a post-hoc sensitivity analysis, we re-ran the final model for physician visits excluding the first month of life, when some visits were likely related to resolving a positive screen as a false positive. This 1-month period was based on the clinical experience of metabolic physicians in Ontario. Analyses were performed using SAS® version 9.3 (SAS Institute, North Carolina, USA).

## Results

### Study population

Forty-three children had false positive newborn screening results for MCADD during the study period. The primary comparison cohort consisted of 545,355 children. The matched comparison cohort included 420 children identified as suitable matches for the false positive children. Children with false positive results for MCADD were more likely to be male, have a birth weight < 2500 g and have a gestational age < 37 weeks relative to the primary comparison group (Table 1). Follow-up time ranged from less than one month to approximately 6 years of age (mean and range: 49 months, < 1 to 67 months in the false positive cohort; 48 months, < 1 to 77 months in the primary comparison group).

**Table 1:** Geographic and sociodemographic characteristics of the study population

	Study Cohort		
	False Positive, *n* (%)	Primary Comparison, *n* (%)	Matched Comparison, *n* (%)
	(*n* = 43)	(*n* = 545 355)	(*n* = 420)
Sex^a,*^			
Male	28 (65.1)	279 638 (51.3)	-
Female	15 (34.9)	265 717 (48.7)	-
Month of Birth			
January – April	19 (44.2)	177 918 (32.6)	142 (33.8)
May – August	8 (18.6)	192 896 (35.4)	134 (31.9)
Sept – December	16 (37.2)	174 541 (32.0)	144 (34.3)
Birth weight^**^			
< 2500 g	6 (14.0)	33 027 (6.1)	28 (6.7)
≥ 2500 g	37 (86.0)	508 466 (93.2)	390 (92.9)
Gestational age^***^			
< 37 weeks	11 (25.6)	42 235 (7.7)	34 (8.1)
≥ 37 weeks	32 (74.4)	489 232 (89.7)	373 (88.8)
Relative income^a^			
‘Lower’	21 (48.8)	232 269 (42.6)	-
‘Higher’	21 (48.8)	310 003 (56.8)	-
Urban–rural status^a,b^			
Rural	< 6 (≤ 11.6)	34 111 (6.3)	-
Urban	38–43 (≥ 88.4)	505 236 (92.6)	-

* *P* < 0.10 for difference in proportion in the false positive cohort versus the comparison cohort

^**^ *P* < 0.05 for difference in proportion in the false positive cohort versus the comparison cohort

^***^ *P* < 0.01 for difference in proportion in the false positive cohort versus the comparison cohort

^a^Counts are not presented for secondary matched comparison group on matched variables

^b^Results are repressed for cell sizes < 6

### Health care visit rates

**Fig. 1:**
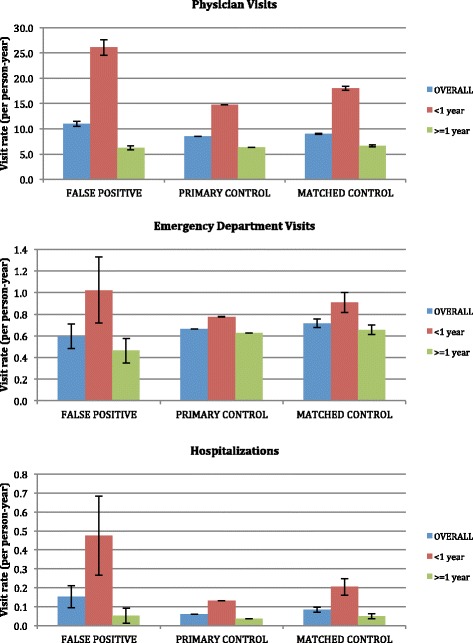
Unadjusted health service visit rates stratified by age at the time of visit (error bars show 95 % confidence intervals)

Children with false positive newborn screening results for MCADD had 1939 recorded physician visits over the follow-up period, with an average unadjusted visit rate of 10.98 physician visits per child per year (Fig. 1). Children in the primary and secondary comparison groups had average rates of 8.44 and 9.00 physician visits per child per year respectively. Those with false positive results for MCADD had a total of 105 ED visits over the follow-up period with an average rate of 0.60 ED visits per child per year; those in the comparison cohorts had averages of 0.66 and 0.72 ED visits per child per year, respectively. Finally, children with false positive newborn screening results had a total of 27 inpatient hospitalizations over the follow-up period with an average rate of 0.15 hospitalizations per child per year. Those in the primary and secondary comparison groups had inpatient hospitalization rates of 0.06 and 0.08 stays per child per year, respectively.

Children in all 3 cohorts had the highest unadjusted rates of physician use, ED visits, and hospitalization rates during their first year of life (Fig. 1). In the first year of life, children with false positive newborn screening results for MCADD had an average of 26.1 physician visits, 1.0 ED visits, and 0.5 hospital admissions per child. These rates decreased to, on average, 6.3 physician visits, 0.5 ED visits, and 0.05 hospitalizations per child per year in subsequent years of age. In the first year of life, those in the primary comparison group had on average 14.7 physician visits, 0.8 ED visits, and 0.1 hospitalizations per child; these rates similarly decreased in subsequent years to an average of 6.3 physician visits, 0.6 ED visits, and 0.04 hospitalizations per child per year. Finally, in the first year of life, children in the matched comparison group had on average 18.0 physician visits, 0.9 ED visits, and 0.2 hospitalizations per child; these rates decreased in subsequent years to an average of 6.6 physician visits, 0.7 ED visits, and 0.05 hospitalizations per child per year.

### Incidence rate ratios for individual health care service types

In the first year of life, after adjusting for potential confounding factors as previously described, children with false positive results for MCADD had a statistically significant higher rate of physician visits (IRR: 1.42 [95 % CI: 1.21–1.67]) and inpatient hospitalizations (IRR: 2.32 [95 % CI: 1.22–4.34]) relative to the primary comparison group; there was no significant difference in the frequency of ED visits (IRR: 1.27 [95 % CI: 0.80–2.03]) (Table 2). There were no statistically significant differences for any of the three service types in the false positive versus primary comparison cohort among children older than 1 year of age. Results were similar for the false positive versus matched comparison cohort, with the exception of ED visits during the first year of life, where children with false positive results had a relatively lower frequency of ED visits (IRR: 0.81 [95 % CI: 0.52–1.27]) (Table 3); this difference was not statistically significant.

**Table 2:** Stratified adjusted models (< 1 year old and ≥ 1 year old) showing incidence rate ratios for the three service types, comparing the false positive cohort with the primary (unmatched) comparison cohort

	Adjusted incidence rate ratio (95 % CI)					
	< 1 year of age			≥ 1 year of age		
	Physician visits	ED visits	Hospitalizations	Physician visits	ED visits	Hospitalizations
**False Positive**	**1.42 (1.21–1.67)**	**1.27 (0.80–2.03)**	**2.32 (1.22–4.34)**	**0.96 (0.77–1.20)**	**0.82 (0.54–1.26)**	**0.68 (0.15–2.25)**
Sex						
Female	0.92 (0.91–0.92)	0.85 (0.85–0.86)	0.76 (0.75–0.77)	0.91 (0.91–0.92)	0.86 (0.85–0.87)	0.78 (0.77–0.80)
Male	Reference	Reference	Reference	Reference	Reference	Reference
Season of birth						
Jan.–Apr.	Reference	Reference	Reference	Reference	Reference	Reference
May–Aug.	1.01 (1.00–1.01)	1.04 (1.03–1.05)	0.98 (0.96–1.00)	0.96 (0.95–0.96)	0.95 (0.95–0.96)	0.94 (0.92–0.97)
Sept.–Dec.	1.00 (1.00–1.01)	1.02 (1.00–1.03)	1.08 (1.06–1.11)	1.00 (0.99–1.00)	1.00 (0.99–1.00)	1.03 (1.00–1.05)
Birth weight						
< 2500 g	1.75 (1.74–1.77)	1.04 (1.01–1.06)	2.14 (2.08–2.21)	1.13 (1.12–1.14)	1.03 (1.01–1.05)	1.59 (1.51–1.66)
≥ 2500 g	Reference	Reference	Reference	Reference	Reference	Reference
Gestational age						
< 37 weeks	1.57 (1.56–1.58)	1.23 (1.21–1.26)	2.65 (2.57–2.73)	1.09 (1.09–1.10)	1.17 (1.15–1.19)	1.42 (1.36–1.48)
≥ 37 weeks	Reference	Reference	Reference	Reference	Reference	Reference
Socioeconomic status						
‘Lower’	1.00 (1.00–1.00)	1.23 (1.22–1.24)	1.09 (1.07–1.11)	0.96 (0.96–0.97)	1.12 (1.11–1.13)	1.10 (1.08–1.13)
‘Higher’	Reference	Reference	Reference	Reference	Reference	Reference
Urban-rural status						
Rural	0.79 (0.78–0.79)	2.34 (2.30–2.37)	1.16 (1.13–1.20)	0.73 (0.72–0.73)	2.28 (2.25–2.31)	1.15 (1.11–1.20)
Urban	Reference	Reference	Reference	Reference	Reference	Reference

**Table 3:** Stratified adjusted models (< 1 year old and ≥ 1 year old) showing incidence rate ratios for the three service types (matched comparison group)

	Adjusted incidence rate ratio (95 % CI)					
	< 1 year of age			≥ 1 year of age		
	Physician visits	ED visits	Hospitalizations	Physician visits	ED visits	Hospitalizations
**False Positive**	**1.45 (1.23–1.72)**	**0.81 (0.52–1.27)**	**2.20 (1.45–3.22)**	**1.00 (0.80–1.26)**	**0.81 (0.53–1.27)**	**0.60 (0.22–1.28)**
Season of birth						
Jan.–Apr.	Reference	Reference	Reference	Reference	Reference	Reference
May–Aug.	1.03 (0.94–1.13)	1.07 (0.86–1.32)	0.73 (0.53–1.02)	0.97 (0.86–1.09)	1.07 (0.87–1.33)	1.67 (1.20–2.34)
Sept.–Dec.	1.04 (0.95–1.13)	1.13 (0.91–1.41)	1.03 (0.77–1.39)	1.04 (0.92–1.16)	1.11 (0.90–1.38)	1.29 (0.90–1.86)
Birth weight						
< 2500 g	1.64 (1.38–1.96)	1.26 (0.85–1.90)	1.46 (0.96–2.21)	1.18 (0.95–1.49)	1.27 (0.86–1.91)	1.60 (1.00–2.52)
≥ 2500 g	Reference	Reference	Reference	Reference	Reference	Reference
Gestational age						
< 37 weeks	1.72 (1.46–2.02)	1.12 (0.77–1.65)	3.07 (2.08–4.43)	1.21 (0.99–1.50)	1.13 (0.78–1.65)	2.18 (1.38–3.34)
≥ 37 weeks	Reference	Reference	Reference	Reference	Reference	Reference

When the age-stratified models were restricted to children with term births (Table 4), the results were similar to those reported for all births (Table 2). In the first year of life, term children with false positive screening results for MCADD had a statistically significant higher rate of physician visits (IRR: 1.35 [95 % CI: 1.13–1.63]) compared to term children with negative screening results. While there was no statistically significant difference in hospitalization rates in the two cohorts among term children less than 1 year of age, the estimated IRR (IRR: 2.32 [95 % CI: 0.87–5.65) was virtually identical to the IRR from the model for all births (IRR: 2.32 [95 %: 1.22–4.34]). No statistically significant differences were observed for health services use among term children aged one year and older. Finally, when we re-examined physician visits over the first year of life in the entire cohort to exclude the first month of life, children with false positive results still experienced higher rates of physician care relative to those in the primary comparison group (IRR: 1.31 [95%CI: 1.11–1.57]).

**Table 4:** Stratified adjusted models (< 1 year old and ≥ 1 year old) showing incidence rate ratios for the three service types for term infants only, false positive cohort versus primary (unmatched) comparison cohort

	Adjusted incidence rate ratio (95 % CI)					
	< 1 year of age			≥ 1 year of age		
	Physician visits	ED visits	Hospitalizations	Physician visits	ED visits	Hospitalizations
**False Positive**	**1.35 (1.13–1.63)**	**1.46 (0.87–2.51)**	**2.32 (0.87–5.65)**	**0.92 (0.72–1.19)**	**0.97 (0.60–1.55)**	**0.65 (0.10–2.60)**
Sex						
Female	0.92 (0.91–0.92)	0.86 (0.85–0.87)	0.75 (0.74–0.77)	0.86 (0.86–0.87)	0.86 (0.85–0.87)	0.78 (0.76–0.80)
Male	Reference	Reference	Reference	Reference	Reference	Reference
Season of birth						
Jan.–Apr.	Reference	Reference	Reference	Reference	Reference	Reference
May–Aug.	1.01 (1.00–1.01)	1.04 (1.03–1.05)	0.98 (0.96–1.01)	0.96 (0.95–0.96)	0.95 (0.95–0.96)	0.94 (0.91–0.96)
Sept.–Dec.	1.00 (1.00–1.00)	1.02 (1.01–1.03)	1.09 (1.07–1.12)	1.00 (0.99–1.00)	1.00 (0.99–1.01)	1.02 (0.99–1.05)
Birth weight						
< 2500 g	1.39 (1.38–1.41)	1.08 (1.04–1.11)	1.88 (1.78–1.99)	1.12 (1.10–1.13)	1.02 (1.00–1.05)	1.60 (1.50–1.71)
≥ 2500 g	Reference	Reference	Reference	Reference	Reference	Reference
Socioeconomic status						
‘Lower’	1.00 (1.00–1.00)	1.22 (1.21–1.24)	1.10 (1.07–1.12)	0.97 (0.96–0.97)	1.12 (1.11–1.13)	1.10 (1.08–1.13)
‘Higher’	Reference	Reference	Reference	Reference	Reference	Reference
Urban-rural status						
Rural	0.78 (0.78–0.79)	2.37 (2.33–2.41)	1.21 (1.16–1.25)	0.73 (0.72–0.73)	2.30 (2.27–2.33)	1.16 (1.11–1.21)
Urban	Reference	Reference	Reference	Reference	Reference	Reference

## Discussion

In this population-based study, after adjustment for potential confounders, children with false positive newborn screening results for MCADD experienced a statistically significant higher frequency of physician visits and hospitalizations, but not ED visits, as compared to screen negative comparison groups in the first year of life. Our findings are consistent with a Beijing study that identified a higher rate of parent-reported hospitalizations in the first 6 months of life among children with false positive versus negative newborn screening results for a range of metabolic disorders [7]. However, an American study, also focused on metabolic disorders, found no statistically significant differences in parent-reported health care use among infants with false positive versus negative screening results [3–5]. The only previous study relying on administrative records, rather than parent reports, involved a US Medicaid population and focused on false positive results for metabolic, endocrine, or hematologic disorders [10]. In that study the authors identified no difference in health care use among children with false positive versus negative screening results, with the exception of preterm infants, among whom false positive results were associated with a higher rate of acute outpatient visits [10]. However, the authors hypothesized that the parental psychosocial response may differ for different screened diseases [10]. To our knowledge, ours is the first disease-specific study to compare health services use in these populations.

While the psychosocial consequences of a false positive newborn screening test are short-term for the majority of parents, studies suggest that a small proportion continue to experience anxiety following diagnostic testing [30, 31]. Such anxiety, or misunderstanding of the diagnostic result, could alter parental perceptions of a child’s vulnerability to illness, similar to the well-documented phenomenon of the “vulnerable child syndrome” [32]. As a result, such families may seek health care at an increased frequency for their child. This is one potential explanation for our findings with respect to physician visits under 1 year of age. However, it is unlikely to explain the increased hospitalizations under one year of age in the false positive versus comparison group, particularly in the absence of a similar increase in ED visits. Given the rarity of MCADD and likely lack of experience with the disorder for most physicians, physician perceptions may also have played a role. For example, this may have resulted in a greater likelihood of hospital admission among infants with false positive results presenting to an ED, particularly if accompanied by parents experiencing anxiety or confusion about the nature of the result.

Another potential explanation for our findings that we considered is that the increase in physician visits during the first year of life might reflect supplementary health service visits related to confirmatory diagnostic testing for the positive newborn screening result. However, after excluding visits during the first month of life, when the vast majority of diagnostic care would occur for children with false positive results in Ontario, that cohort still experienced a higher rate of physician visits in the entire first year of life relative to screen-negative controls.

A further possible explanation for the higher rate of the use of certain health services in the first year of life in children with false positive results is a residual confounding effect of premature birth. We adjusted for gestational age and birth weight in our multivariable analyses, since children with false positive results for MCADD are more likely to be of lower gestation and birth weight [10-12]. However, due to our small sample size, we dichotomized both birth weight and gestational age rather than using smaller categories as has been recommended [33, 34]. To investigate a role for residual confounding by gestational age, we restricted the analysis to children with gestational age ≥ 37 weeks. In that analysis, the results for physician visits were similar to those in the main analysis and the results for hospitalizations yielded the same effect size; this suggests that residual confounding by gestational age does not explain our findings.

Finally, it is possible that physiological differences aside from gestational age and birth weight may be associated with the biochemical markers used in the screening test and may influence health care use during the first year of life. For example, we did not have information about underlying illnesses aside from those that are part of the newborn screening panel. This potential explanation for our findings is supported by a study that identified a higher prevalence of low birth weight and/or time spent in a neonatal intensive care unit in infants with elevated screening markers for MCADD [35]. A limitation of using health care administrative datasets is the lack of both clinical detail and family-reported information. As a result, we were unable to directly investigate the role of clinical characteristics such as underlying illness and, similarly, we could not directly study parental or physician perceptions of infants’ health. Further research is needed to further explore these potential explanations. Should such research confirm a psychosocial effect leading to higher health service use, our findings support the use of strategies shown to mitigate parental anxiety in the face of false positive findings, including effective education and provider communication during confirmatory follow-up [30, 36].

## Conclusions

Infants with false positive newborn screening results for MCADD may experience higher health care use in the first year of life as a result of parental and physician perceptions of their health status or unmeasured confounding. More research is needed to investigate competing explanations and to directly study the psychosocial impact of false positive findings. Understanding the impact of false positive newborn screening results can help to inform newborn screening programs, and health care providers involved in such programs, in designing support and education to benefit families of children receiving positive results. Specifically, if some parents or non-metabolic specialist health care providers have misperceptions about the health of children with false positive newborn screening results, there may be a need for further parental and provider education about the clinically benign status of such results; and for additional counselling services for parents who remain concerned. This is particularly important as additional disorders are added to expanded screening panels, leading to important clinical benefits for children with rare treatable disorders but also a higher frequency of false positive findings.
